# In-lab assessment of a ice hockey helmet-integrated sensor fusion framework for torque estimation: A proof-of-concept study

**DOI:** 10.1016/j.jsampl.2026.100160

**Published:** 2026-09-25

**Authors:** Dario Sciacca, Anisoara Ionescu

**Affiliations:** Signal Processing Laboratory 5, Swiss Federal School of Technology (EPFL), 1015 Lausanne, Switzerland

**Keywords:** Instrumented helmet, Force sensor, Torque, Head impact biomechanics, Inertial Measurement Units

## Abstract

This study presents a proof-of-concept ice hockey helmet–integrated system for characterizing in-lab rotational loading, consisting of a single Inertial Measurement Unit (IMU) and custom soft foam force sensors. A total of 72 controlled pendulum impacts (33 J and 79 J) were delivered to a helmeted Anthropomorphic Test Device (ATD) at side and crown locations. A custom algorithm detected impacts and estimated torque by combining IMU-derived directions with force magnitudes. Results showed successful discrimination between conditions, with crown impacts producing lower torque. Recognizing torque as a relevant metric may improve monitoring and prevention of brain injury in contact sports.

## 1 Introduction

Injuries resulting from head rotational motion remain a major concern in contact sports. Rapid rotation of the skull can produce relative displacement of the brain within the cranial cavity, generating shear stresses and tensile strains within brain tissue and potentially leading to diffuse axonal injury [1]. As a result, head rotational kinematics is widely considered key contributor to concussion risk [1].

Beyond commonly used global head kinematic measures, the forces and loading conditions experienced by the head during impact may provide additional insight into concussion injury mechanisms [2]. In particular, torque describes the tendency of a force to induce rotation and captures both its magnitude and point of application. Torque is related to angular acceleration through the moment of inertia, expressed as $\vec{\tau }=I\vec{\alpha }$ [3]. In head biomechanics, this relationship is influenced by factors such as neck strength, posture, and muscle activation, which modify the effective rotational inertia of the head–neck system. Increased activation of the neck and torso may reduce peak head rotation: the eccentric action of the cervical muscles decelerates the displaced head while recruiting a portion of the torso's mass to contribute to resisting the impact [4]. In this context, greater neck strength has been associated with reduced concussion risk [5].

Motivated by the relevance of rotational head loading and its potential implications for traumatic brain injury risk [6], this study presents a proof-of-concept helmet-integrated system combining a single Inertial Measurement Unit (IMU) with soft foam force sensors to estimate impact torque. In contact sports such as ice hockey and American football, head impact measurement has relied predominantly on inertial sensing [7], while force sensing in helmets has been used primarily to characterize helmet fit or foam performance [8,9]. However, the integration and complementary use of kinematic and force sensing remain limited. The aim of this work is to evaluate whether combining these measurements can provide meaningful discrimination between controlled impact loading conditions in a laboratory setting, comparing two representative impact locations, the side and the crown, selected for their differing kinematic response.

## 2 Methods

### 2.1 Measurement setup

Controlled impact tests were conducted using a Hybrid III 50th percentile male Anthropomorphic Test Device (ATD) (Diversified Technical Systems, DTS, United States), mounted on a linear sliding carriage system (Biokinetics & Associates LTD, Canada). A pendulum impactor (mass 6.7 kg) delivered impacts at two energy levels (33 J and 79 J), corresponding to pendulum release angles of 30° and 50°. These angles correspond to impact velocities of approximately 3 and 4.6 m/s, respectively, which fall within the range typically associated with sub-concussive and concussive head impacts in ice hockey [10]. Impacts were applied at two anatomical locations: left/right side and crown (72 total number of impacts). Crown impacts were achieved by tilting the headform forward (∼30°) to align the neck axis with the impact direction. An ice hockey helmet (RE-AKT 150, size M, Bauer, Canada) was instrumented with an IMU (MindMaze, Switzerland) comprising a triaxial accelerometer (ADXL375, Analog Devices, ±200 g) and a gyroscope (LSM6DSOX, STMicroelectronics, ±2000°/s), placed on the back of the helmet, which was locally modified to accommodate it. The helmet also included a custom force sensor system (Bearmind SA, Switzerland), consisting of four pads made of three conductive silicone layers enclosed between two foam layers (soft, 5 mm, and hard, 2.4 mm). Sensor geometry was adapted to the helmet pads, and the foam combination provided dual sensitivity: below 25 N and 25–1000 N for lighter and intense impacts, respectively (more details are provided in Supplementary material, Section S1).

All signals were recorded using a shared data acquisition system. Acceleration and force signals were sampled at 1024 Hz, while angular velocity was sampled at 512 Hz. In particular for the IMU, these sampling rates were considered appropriate based on the signal bandwidths adopted in previous studies using helmet-mounted systems [11].

### 2.2 Data processing and analysis

IMU data were transformed into a helmet-based Functional Frame (FF) to ensure consistency across trials, defined as X_FF (antero-posterior), Y_FF (axial), and Z_FF (medial-lateral), through a dedicated calibration procedure [12]. Impact events were detected using combined thresholds on linear acceleration and angular velocity [12], while force-based detection relied on the resultant force across all pads exceeding a 70 N threshold, determined from preliminary laboratory testing. For the force sensors, a calibration algorithm was implemented to minimize the effect of individual sensor variability for the different locations (see Section S2 in Supplementary material for details).

Impact direction estimation was performed using a Principal Component Analysis (PCA)-based algorithm applied to the IMU signals [12]. The input signal was selected according to the impact configuration, which was prescribed by the test protocol: angular velocity for side impacts, and linear acceleration for crown impacts, due to the reduced rotational dominance in this configuration. In each case, PCA extracted the dominant vector of the selected signal, which was used in the torque estimation.

Force sensor data were used to provide supplementary information on impact location. For each pad, the relative contribution was computed as:

(1) $$C_{p,l}=\frac{F_{p,l}}{F_{\max,l}}\times 100\%$$

where $C_{p,l}$ is the contribution of the pad p at location l, $F_{p,l}$ is the average peak force for the pad p at location l, and $F_{\max,l}$ is the global peak force recorded at the location l across all the pads.

The torque $\vec{\tau }$ was then estimated using the following equation:

(2) $$\vec{\tau }=\vec{r}\times \vec{F}=\left|r\right|\left|F\right|\sin\left(\;\gamma \;\right)$$

Where $\vec{r}$ is the distance vector from the head center of rotation to the point where the force is applied, $\vec{F}$ is the resultant force vector and γ is the angle between $\vec{r}$ and $\vec{F}$. For more details on the definition of the *r* vector, please see Section S3 in Supplementary material.

Torque (Equation (2)) was computed using the peak resultant force from the four pads and the angle γ between $\vec{r}$ and the main IMU-derived impact direction vector. An overview of the implemented pipeline is shown in Fig. 1.

**Fig. 1:**
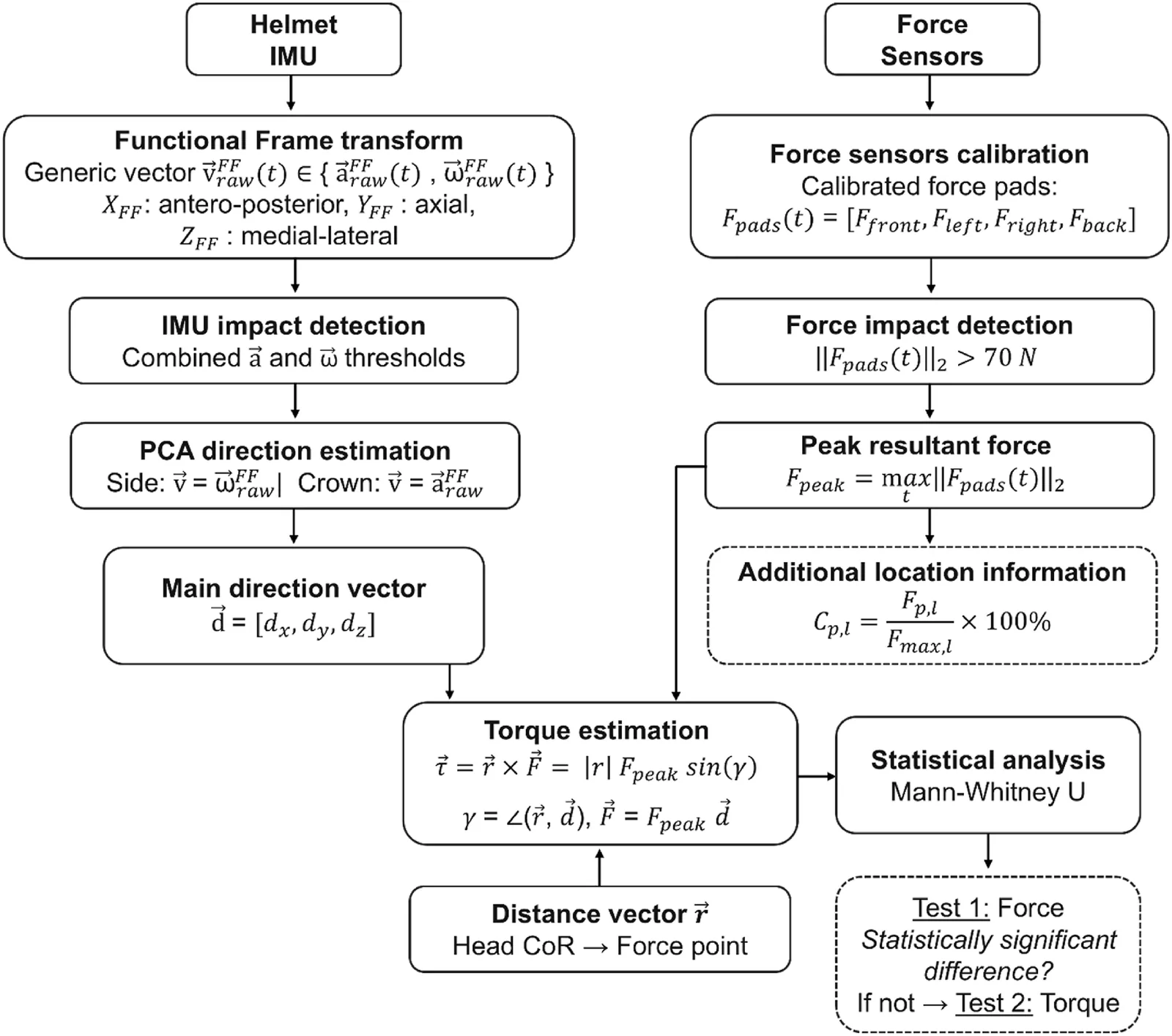
**–** Overview of the data processing and analysis pipeline. Helmet IMU data are transformed into the helmet-based Functional Frame (*FF*), where a generic vector $\vec{v}$ represents either angular velocity for side impacts or linear acceleration for crown impacts. PCA is used to estimate the main direction vector $\vec{d}$. Force sensor data are calibrated, used for force-based impact detection, and summarized as the peak resultant force *F*_*peak*_, with pad contributions *C*_*p,l*_ providing supplementary impact location information. Torque is estimated from the distance vector $\vec{r}$, the IMU-derived direction $\vec{d}$, and *F*_*peak*_. Abbreviations: FF = Functional Frame; CoR = Center of Rotation.

Differences between side and crown impacts were evaluated using Mann–Whitney U tests (α = 0.05). Force magnitudes were first compared to assess potential confounding effects; torque was subsequently tested if no significant force differences were present.

## 3 Results

IMU-based direction analysis confirmed clear separation between impact locations. Side impacts were primarily characterized by lateral motion components, while crown impacts exhibited stronger anterior–posterior alignment (Table 1).

**Table 1:** Impact direction analysis for crown and side impacts at 30° and 50°. The average angle between the estimated impact directions (using the IMU data) and the *r* vector are illustrated, as well as the average direction vector for each impact configuration.

Location	Angle [°]	Avg. Angle (±std)	Avg. direction vector
Crown	30	132.87 (2.98)	[−0.96, −0.24, 0.04]
Crown	50	129.92 (5.97)	[−0.97, −0.19, 0.01]
Side	30	114.92 (4.82)	Right: [−0.1, −0.09, −0.99] Left: [0.06, 0.1, 0.99]
Side	50	119.89 (6.65)	Right: [0.03, −0.03, −1] Left: [0.1, 0.13, 0.98]

Force sensors distributions further confirmed spatial consistency, with each impact location predominantly activating the nearest corresponding pad (see Section S4 in Supplementary material).

The mean angle between the force direction and the *r* vector was consistently higher for crown impacts compared to side impacts, 132.87° vs 114.92° and 129.92° vs. 119.89° for 30° and 50° of pendulum arm angle, respectively. Additionally, the variability was consistently lower, although the differences between the two locations remain below 2° for the two intensities.

The results of the Mann–Whitney U tests, comparing both the peak resultant force and the corresponding torque measurements between the two locations, indicated that force magnitudes did not differ significantly between impact locations at either energy level (p = 0.40 for 30°, p = 0.69 for 50°). In contrast, torque values showed statistically significant differences between side and crown impacts for both conditions (p < 0.001), demonstrating clear separation between configurations. The summary statistics and corresponding boxplots are shown in the Section S5 of the Supplementary material.

## 4 Discussion

For the IMU, the assessment of impact directions (Table 1) confirmed correct location identification in accordance with the FF, with side impacts mainly expressed along the z-direction and crown impacts along the x-direction. Force sensor results similarly indicated accurate spatial mapping, with each impact location predominantly activating its corresponding pads.

Differences in torque between crown and side impacts can be interpreted in terms of head orientation during loading, as reflected by the observed angle differences. The reduced y-component at the crown leads to a larger mean angle relative to the reference *r* vector, indicating a more direct alignment of the applied force with the neck axis.

Statistical analysis further supports these biomechanical distinctions. Torque exhibited significant location-dependent differences at both 30° and 50° impact intensities, whereas force magnitudes showed no significant variation across locations. This suggests that force magnitude alone does not explain the observed differences, and that torque variations are primarily driven by differences in impact direction.

Several limitations should be considered for the present study. First, the *r* vector was assumed constant and was manually defined, which may not capture small variations due to helmet–head decoupling during impact. Although alternative approaches, such as motion capture system, could improve accuracy, this assumption may be reasonable given the millisecond-scale duration of the loading phase, during which the force application point is unlikely to vary substantially. In addition, future work could improve torque estimation accuracy by resolving spatial force distributions at the pad level to better define the effective point of application. Incorporating full force time histories, rather than peak values alone, may also be beneficial.

A further limitation concerns the use of a single IMU positioned at the posterior aspect of the helmet, a location selected to shield the sensor from direct impact and reduce the risk of damage. Although impact loading is transmitted through the helmet shell and padding, the IMU measurements, and the accelerometer signal in particular, may still be affected by the impact location relative to the sensor. Future work should examine multiple sensor locations to characterise this spatial dependence.

This proof-of-concept study is also limited by sample size as well as the restricted set of impact configurations, designed to represent two distinct rotational scenarios, and would require broader testing to enhance generalizability. Finally, absolute torque values were not validated against an external reference. Nevertheless, the main objective of this preliminary study was to evaluate the discriminative capability of combined kinematic and force data in controlled head impacts, thereby providing a foundation for future work on rotational loading-based frameworks.

## Disclosure of interest

The authors report there are no competing interests to declare.

## Data availability

The data are available from the corresponding author upon reasonable request.

## Author’s contributions

DS contributed to study design, data collection, data processing, visualization, and manuscript writing (original draft and revisions). AI contributed to study design and manuscript review. All authors have read and approved the final manuscript.

## Funding

This research did not receive any specific grant from funding agencies in the public, commercial, or not-for-profit sectors.

## Declaration of competing interest

The authors declare that they have no known competing financial interests or personal relationships that could have appeared to influence the work reported in this paper.
