# Supplementary material for: In-lab assessment of a ice hockey helmet-integrated sensor fusion framework for torque estimation: A proof-of-concept study
Source: JSAMS Plus. 2026 Sep 25;8:100160. doi: 10.1016/j.jsampl.2026.100160 (PMC13636071; doi:10.1016/j.jsampl.2026.100160)
Supplement: Supplementary file 1 [file mmc1.pdf]

Supplementary Material

S1. IMU and force sensors system

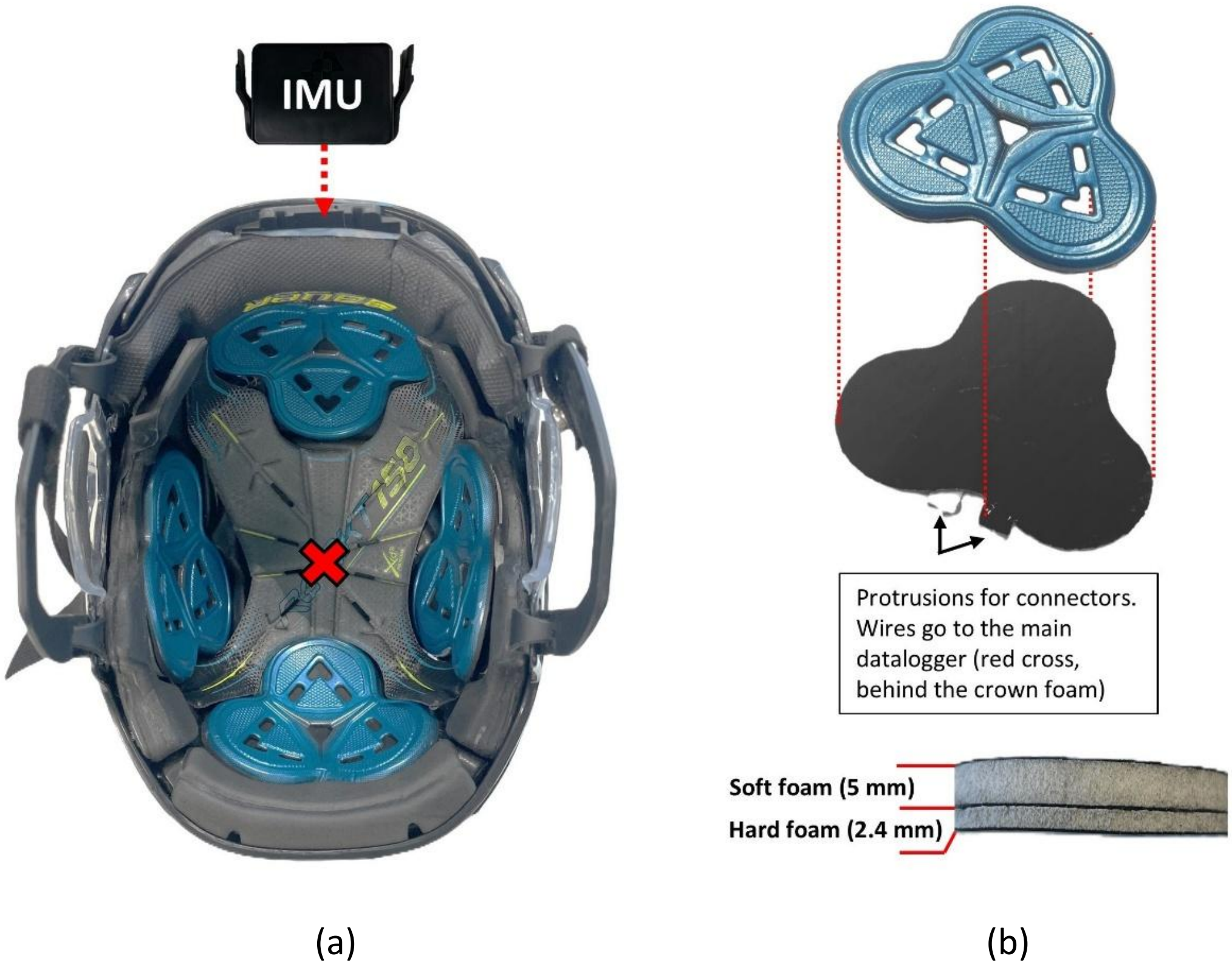

Figure s1 - (a) Top view (inner side) of Bauer helmet equipped with IMU (mounted at the back) and force sensors. The red cross indicates the approximate location of the main datalogger, which is positioned between the liner and the outer shell (not visible in the figure); (b) Detailed illustration of a single force sensing pad.

S2. Force sensors calibration

During preliminary analysis of the side impact data, two distinct clusters emerged based on peak force magnitudes: one below 400 N and one above (Figure s2a). To investigate this further, a pilot study was conducted involving 60 controlled impacts at three energy levels (33 J, 79 J, and 144 J, corresponding to pendulum arm angles 30, 50 and 70 degree, respectively) targeted at the side location of the helmet. The helmet was fitted as tightly as possible to ensure minimal movement during impacts. Notably, all impacts produced forces exceeding 400 N (Figure s3), leading to the hypothesis that force values below 400 N in the original dataset may be associated with factors such as helmet decoupling or suboptimal fit.

Based on this observation, impacts above 400 N in the original dataset were considered representative of the "ideal scenario" for side impacts. These data points were used to fit a linear regression model of peak resultant force as a function of pendulum arm angle, forming the basis for the calibration.

The calibration procedure was as follows (for each angle):

1. The expected (ideal) force value was extracted from the regression line.
2. The original dataset was split into two groups: one with force values <400 N and one with values ≥400 N.
3. For each group, the mean force was computed.
4. The difference between each group's mean and the ideal value was calculated.
5. These differences were then applied as correction factors to adjust the individual force measurements (Figure s2b)

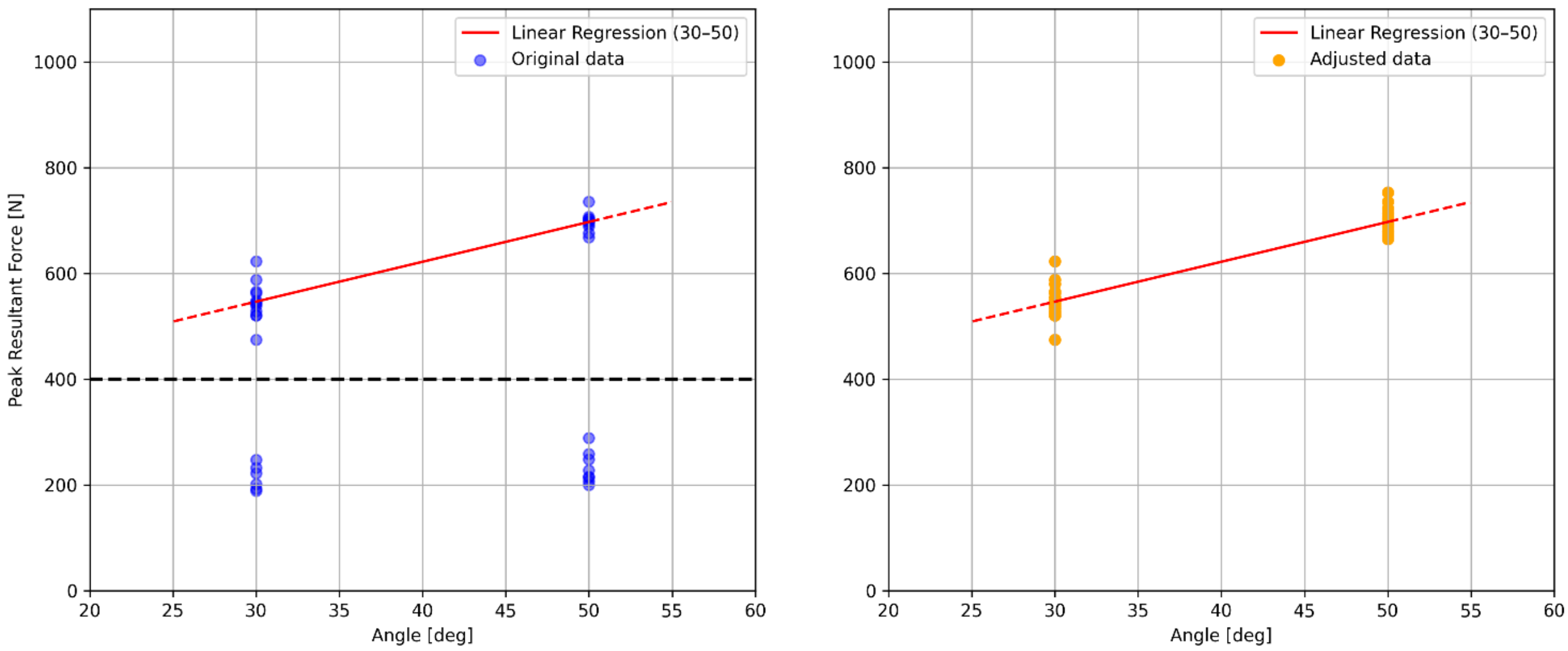

(a) (b)

Figure s2 - Calibration of force sensor data for side impacts (n=36). (a) Original data and regression model (built with force data above 400 N) are shown; (b) Data adjusted with the calibration.

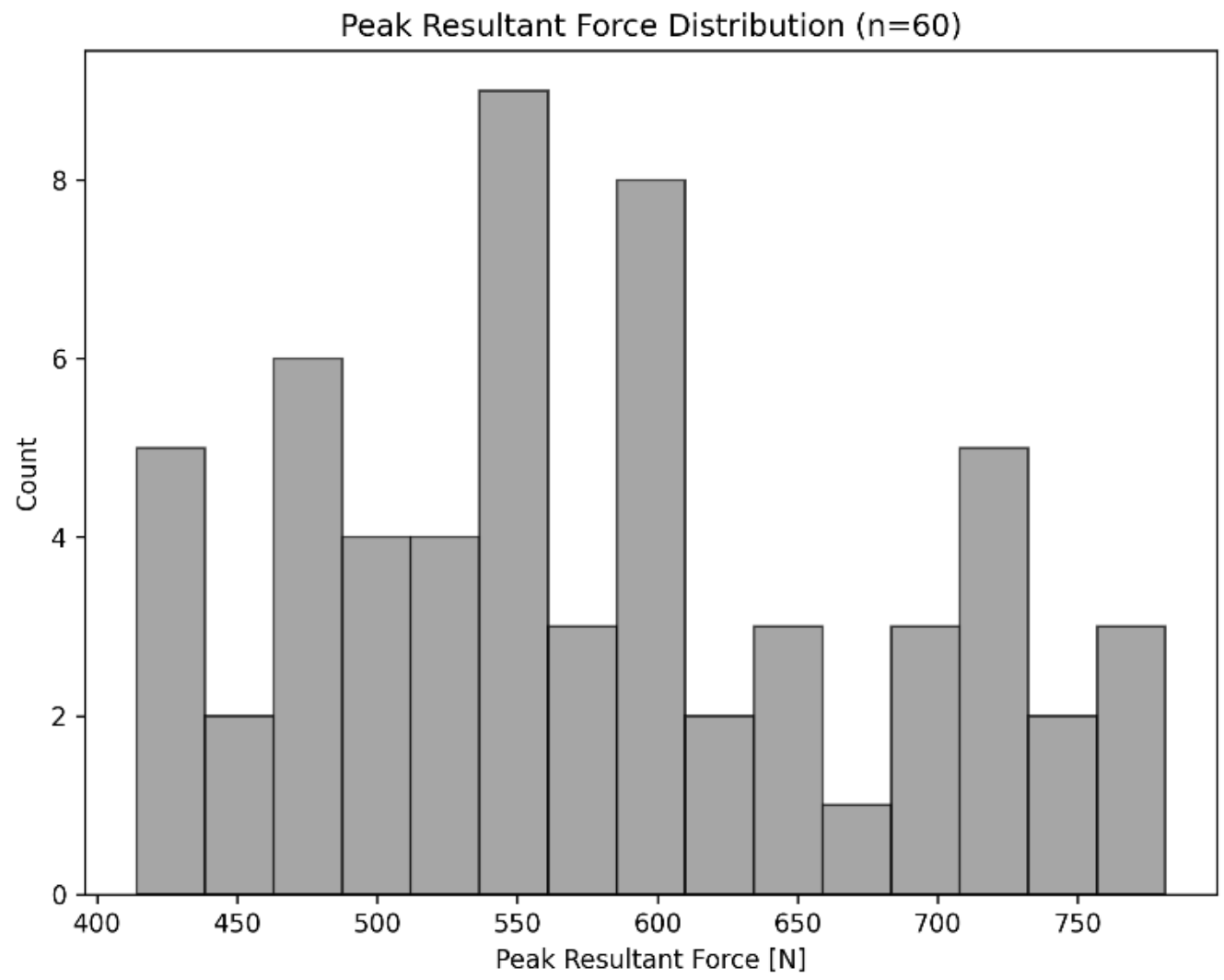

Figure s3 - Distribution of peak resultant force values recorded during the pilot study (n = 60) conducted on the side location.

The same calibration model was subsequently applied to the crown location data using the same adjustment procedure. The side location was used as the calibration reference because, in this configuration, the impact strikes approximately the center of the pad — the closest scenario to the original pad validation conditions.

Examples of force profiles recorded from the four pads for each impact configuration are presented in Figure s3:

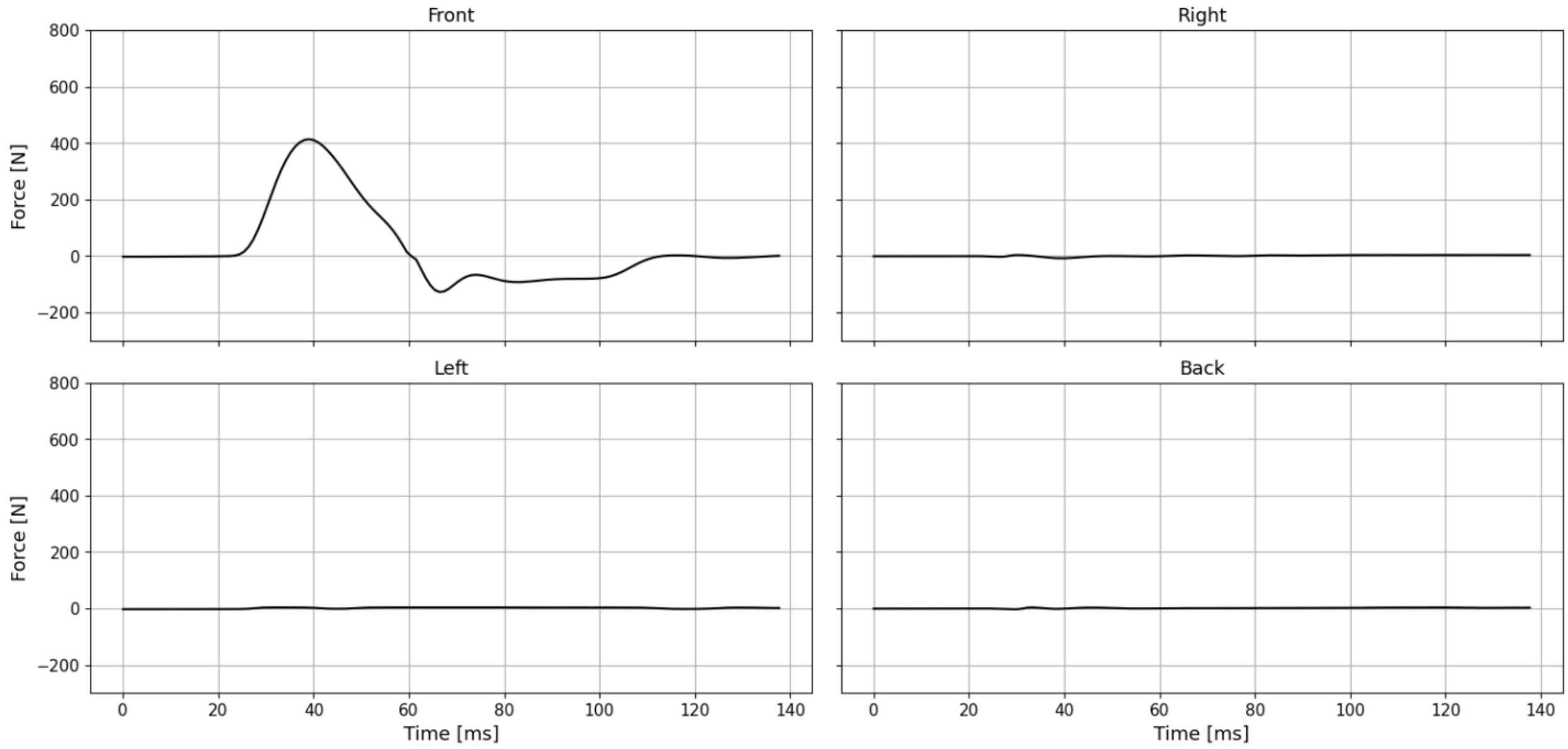

(a) Crown 33 J (30 degree)

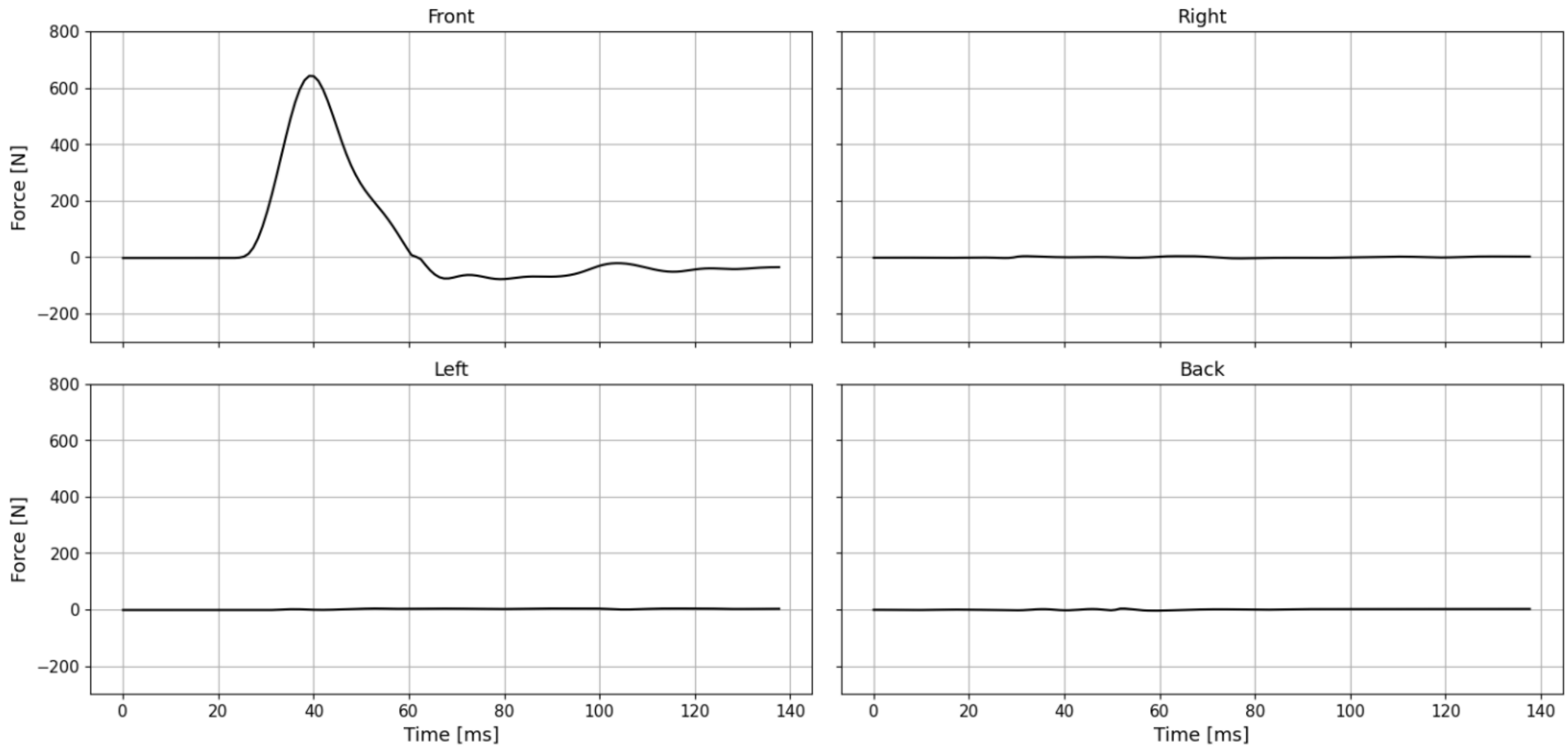

(b) Crown 79 J (50 degree)

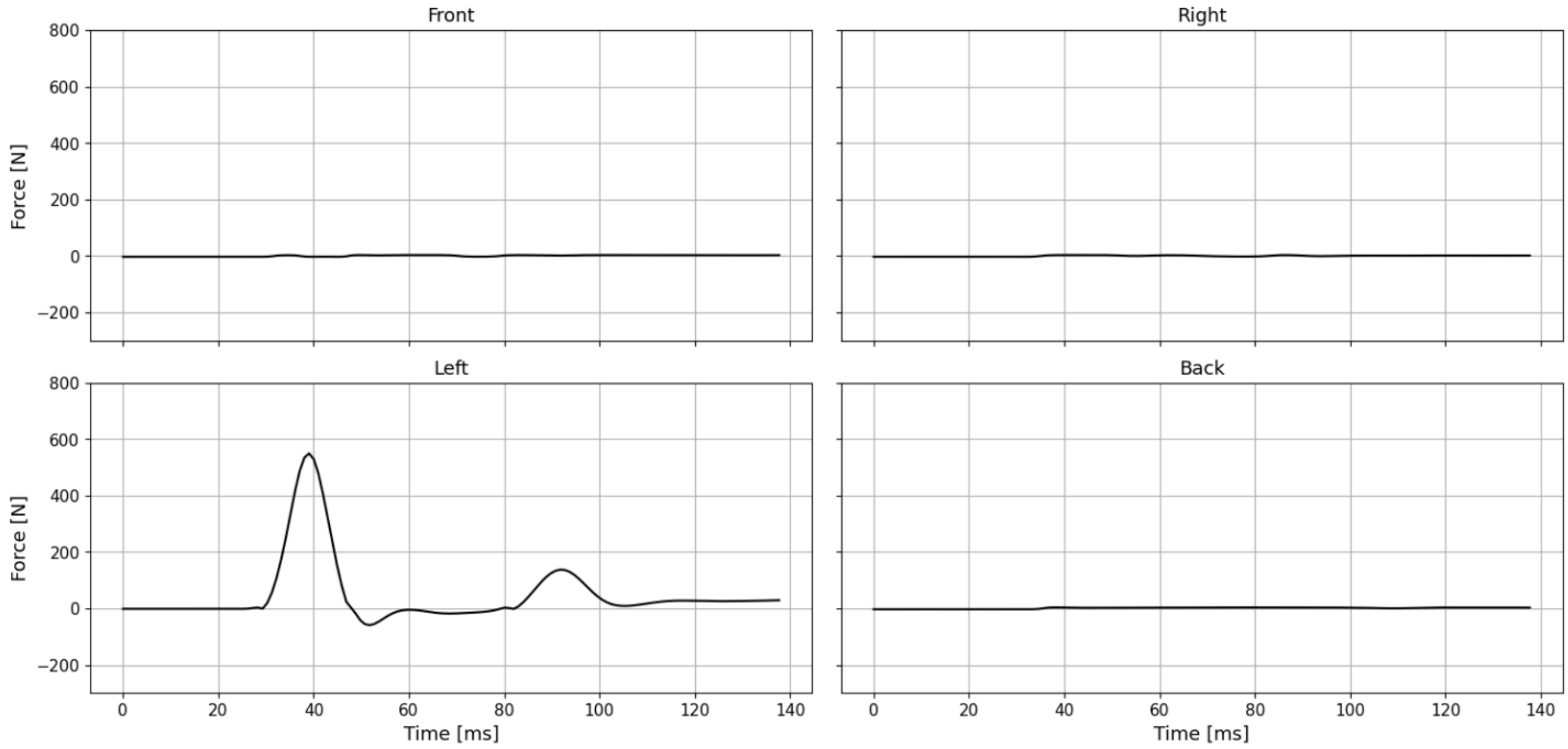

(c) Left Side 33 J (30 degree)

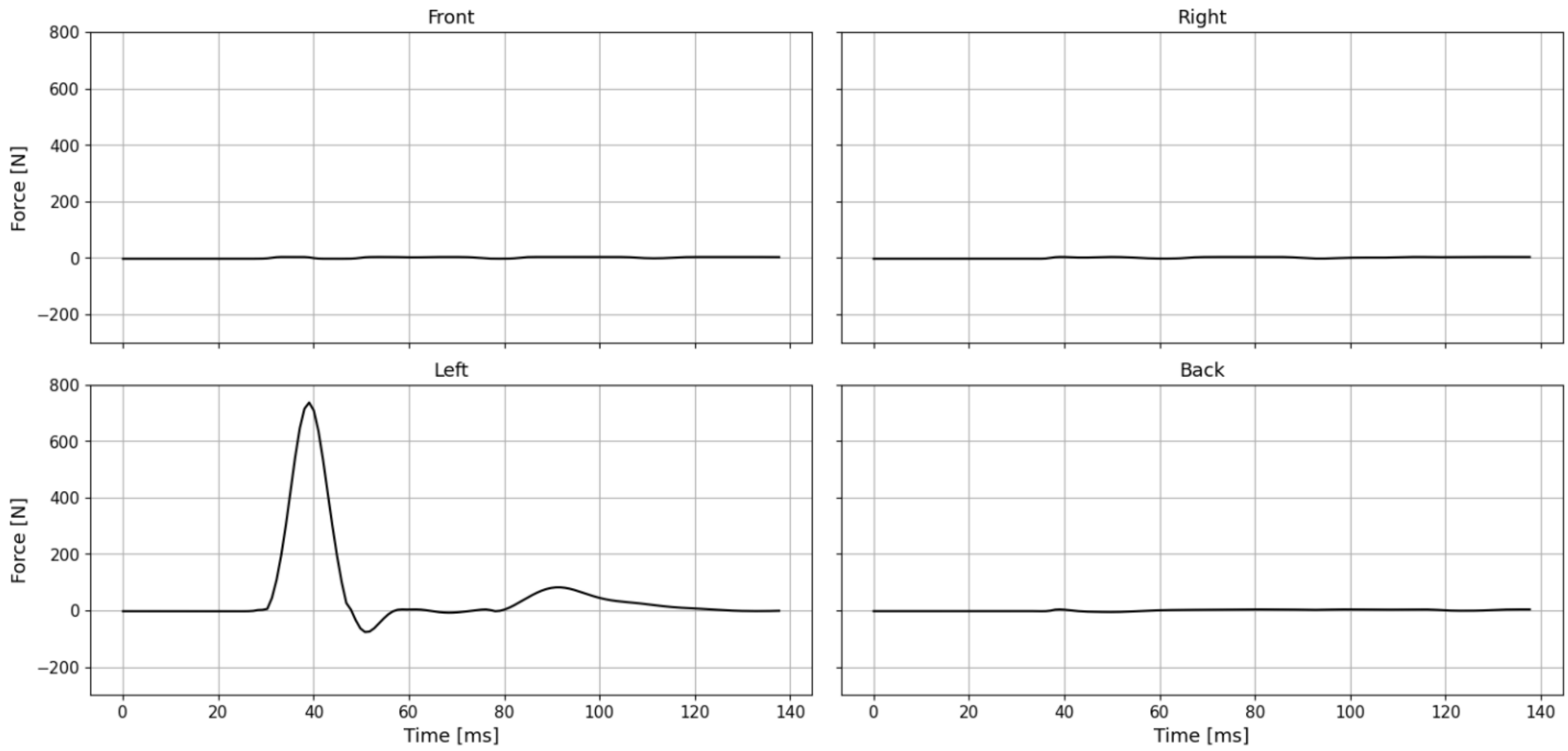

(d) Left Side 79 J (50 degree)

Figure s4 - Examples of force profiles recorded from the four force pads for each impact configuration

S3. Distance Vector Estimation

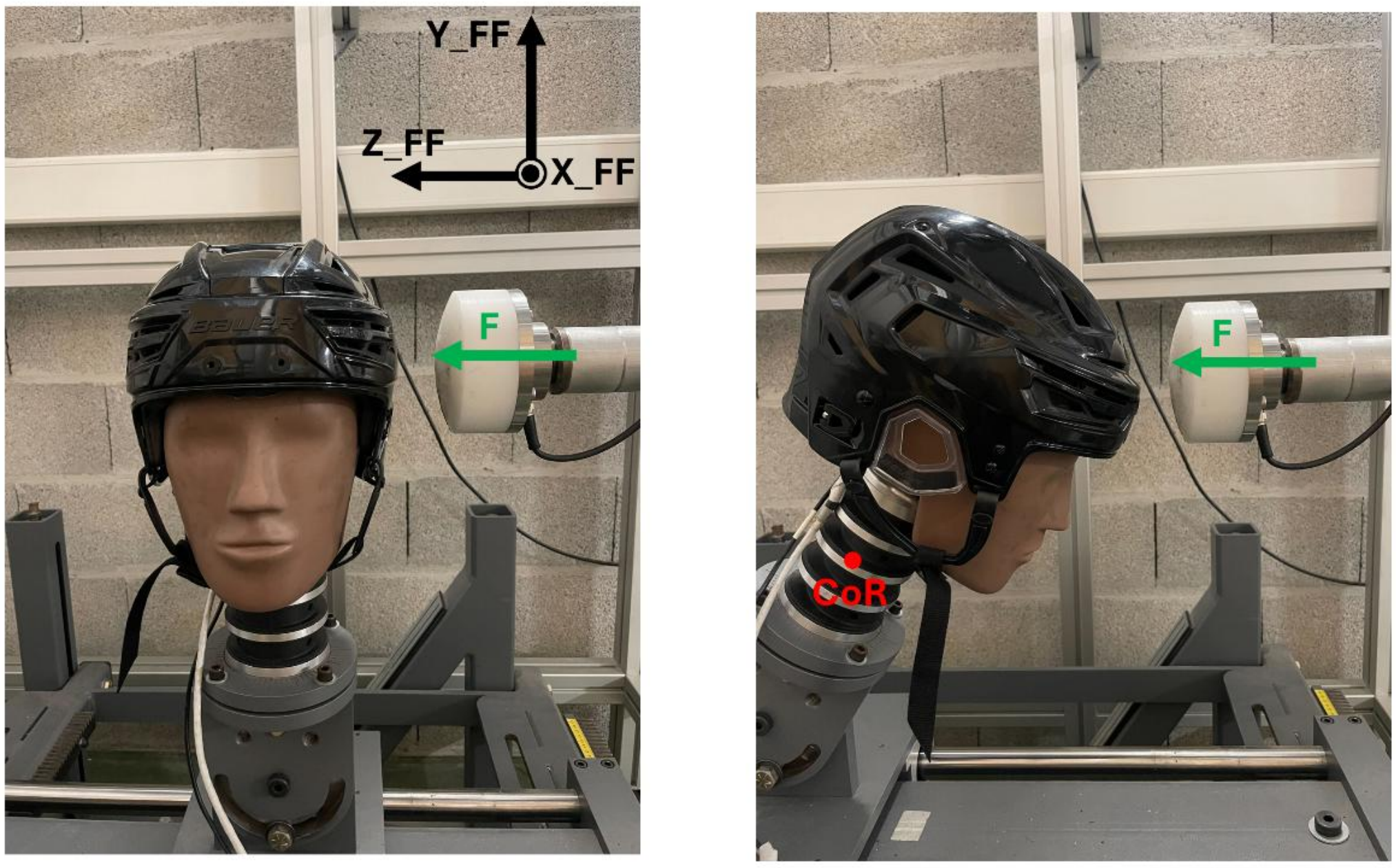

(a) (b)

Figure s5 - Illustration of the Side (a) and Crown (b) impact locations. The direction of the applied force is indicated in green. The Functional Frame (FF) is shown for the Side location, for clarity. The Center of Rotation (CoR) is marked as a red dot for the Crown location, for clarity.

Figure s5 shows that crown impact forces are more aligned with the neck, supporting inclusion of this location to capture distinct rotational responses. The center of rotation (CoR) was defined based on previous studies (Kunin et al., 2007), and the r vector was manually set according to the FF: [0, 0.18, −0.1] m, [0, 0.18, 0.1] m and [0.1, 0.18, 0] m for left side, right side and crown, respectively, assuming the vector to be symmetrical.

S4. Results for Impact Location Estimation

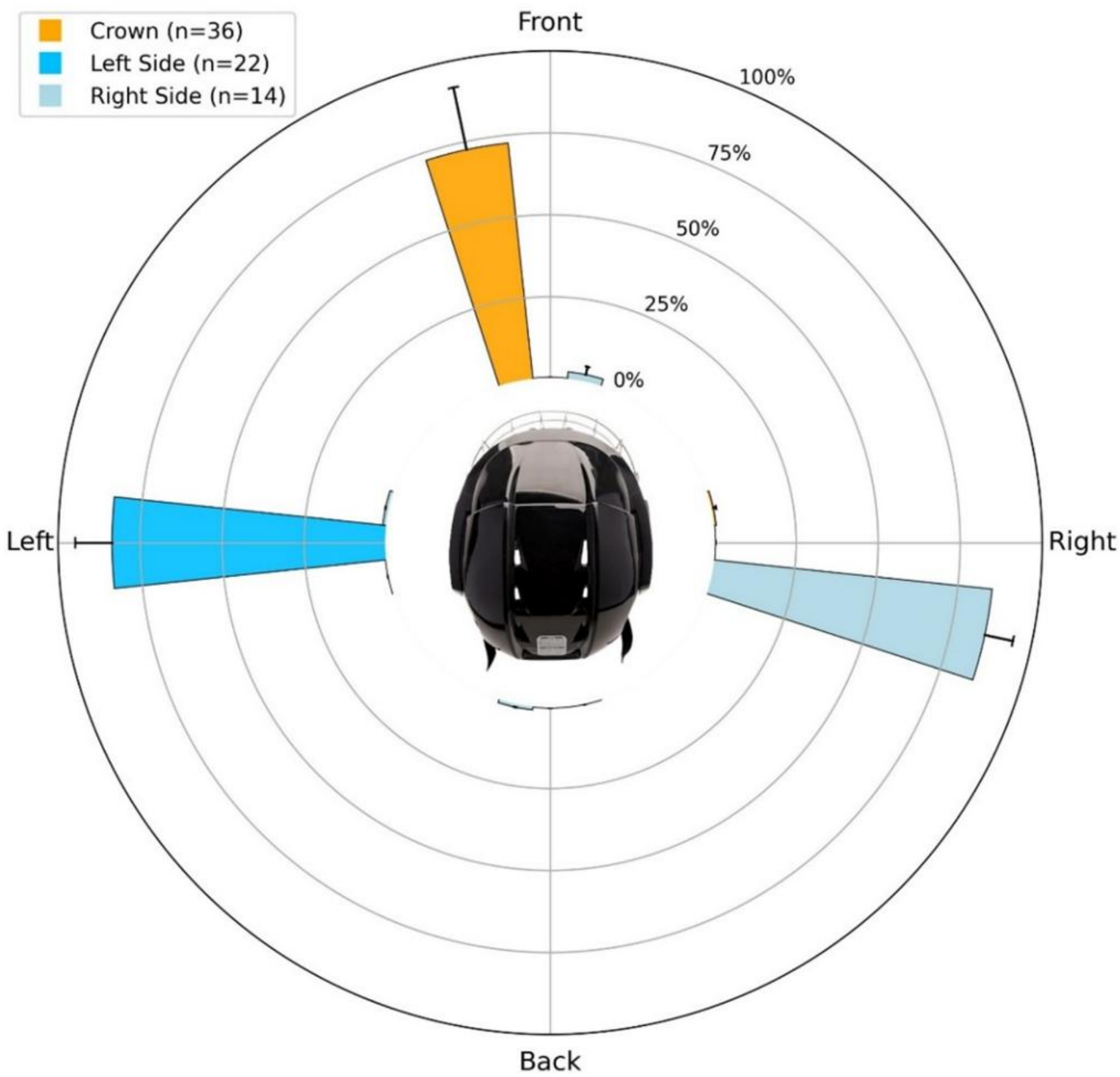

Figure s6 - Polar bar plot (helmet top view) of the average contribution of each pad to the force measurement at each sensor location. Error bars show standard deviation.

S5. Results for Torque discrimination

Table s1 - Summary of p-values for statistical comparisons assessing the similarity in force and torque measurements between groups using the Mann–Whitney U test (significance level of α = 0.05). * indicates statistically significant differences.

Group	Sample size	P-value
Force (30°)	18	0.40179
Force (50°)	18	0.69249
Torque (30°)	18	0.00002*
Torque (50°)	18	0.00095*

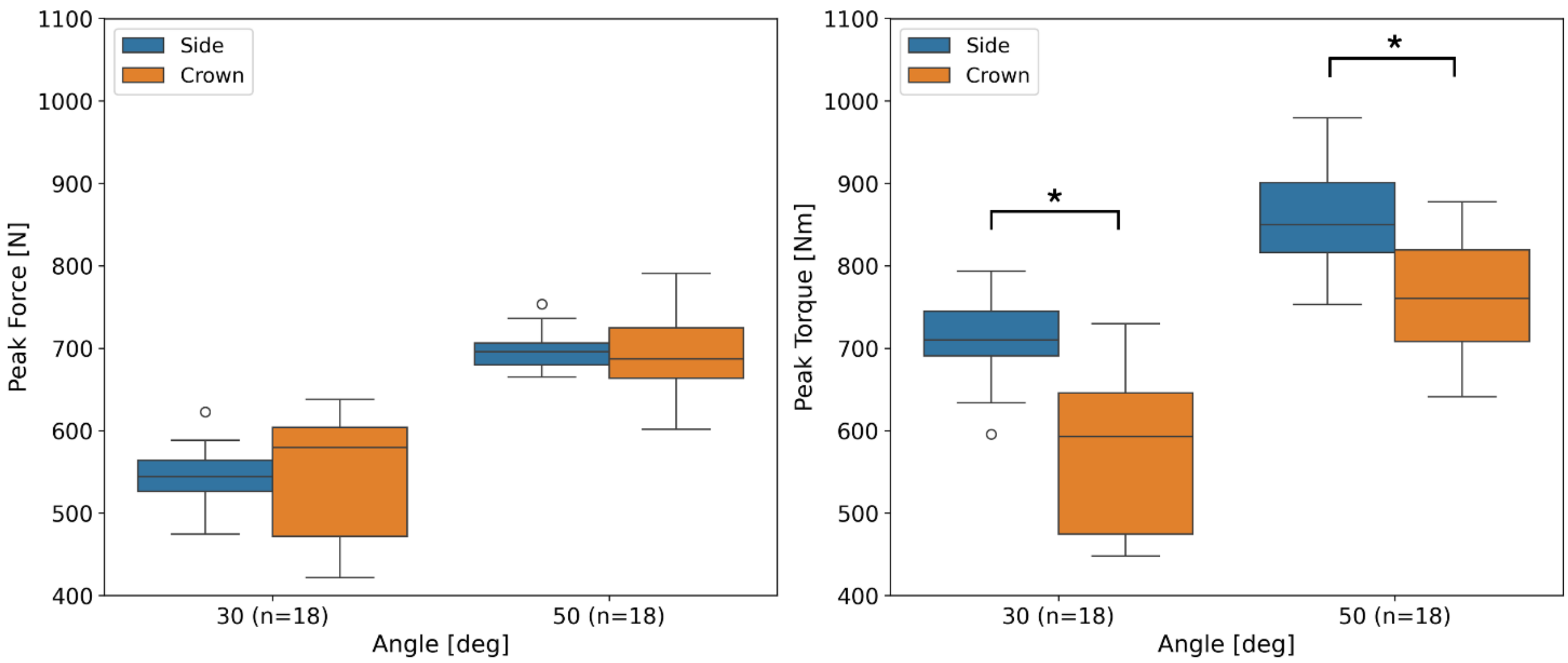

Figure s7 - Boxplots for estimated peak resultant force and corresponding torque. * indicates statistically significant differences

S6. References

Kunin, M. *et al.* (2007) "Rotation axes of the head during positioning, head shaking, and locomotion," *Journal of Neurophysiology*, 98(5), pp. 3095–3108. Available at: https://doi.org/10.1152/jn.00764.2007.
